# High-throughput functional trait testing for bacterial pathogens

**DOI:** 10.1128/msphere.00315-23

**Published:** 2023-09-13

**Authors:** Zachary R. Stromberg, Shelby M. B. Phillips, Kristin M. Omberg, Becky M. Hess

**Affiliations:** 1 Chemical and Biological Signatures Group, Pacific Northwest National Laboratory, Richland, Washington, USA; The University of Texas Medical Branch at Galveston, Galveston, Texas, USA

**Keywords:** bacterial pathogen, high-throughput methods, microbial functional traits, pathogen agnostic detection, pathogenesis

## Abstract

Functional traits are characteristics that affect the fitness and metabolic function of a microorganism. There is growing interest in using high-throughput methods to characterize bacterial pathogens based on functional virulence traits. Traditional methods that phenotype a single organism for a single virulence trait can be time consuming and labor intensive. Alternatively, machine learning of whole-genome sequences (WGS) has shown some success in predicting virulence. However, relying solely on WGS can miss functional traits, particularly for organisms lacking classical virulence factors. We propose that high-throughput assays for functional virulence trait identification should become a prominent method of characterizing bacterial pathogens on a population scale. This work is critical as we move from compiling lists of bacterial species associated with disease to pathogen-agnostic approaches capable of detecting novel microbes. We discuss six key areas of functional trait testing and how advancing high-throughput methods could provide a greater understanding of pathogens.

## OPINION/HYPOTHESIS

Classifying bacterial organisms as pathogens, non-pathogens, and commensals often neglects the magnitude of the host response. Although it has been recognized that all microbes have some pathogenic potential (1), we generally refer to bacterial pathogens as organisms that cause disease in a host. To cause disease, bacterial pathogens use a variety of factors to colonize, evade, and overcome the host response. Classically, Koch’s postulates and molecular Koch’s postulates have been used to understand the pathogenesis and elucidate which factors pathogens use to cause disease (2).

Microbial functional traits are the measurable characteristics that affect organism fitness, performance, and behavior under certain conditions (3 – 5). Functional traits may be produced by the bacterium itself (e.g., toxin production), by the interaction of the bacterium with the host (e.g., cell death), or by the environment (e.g., competition with other microbes). Traits of individual bacteria can be difficult to identify in complex systems and can be transferred between bacteria by horizontal gene transfer, mainly when encoded by plasmids (6). For species within communities, functional traits contain information on environmental constraints, phylogenetic signals, and physiological function (4, 7, 8).

Traditionally, microbiologists analyzed functional traits based on phenotypic tests; however, high-throughput sequencing has enabled extensive efforts to infer functional traits from genomes. Because phenotypic trait testing is laborious and some microorganisms are challenging to grow in culture, data about microbial phenotypic traits are more scarcer than those about genotypic traits. Therefore, to thoroughly characterize bacterial pathogens, we must better understand how genotype translates to a functional trait and consider enzymes and morpho-physio-phenological traits as functional traits (9).

**Fig 1:**
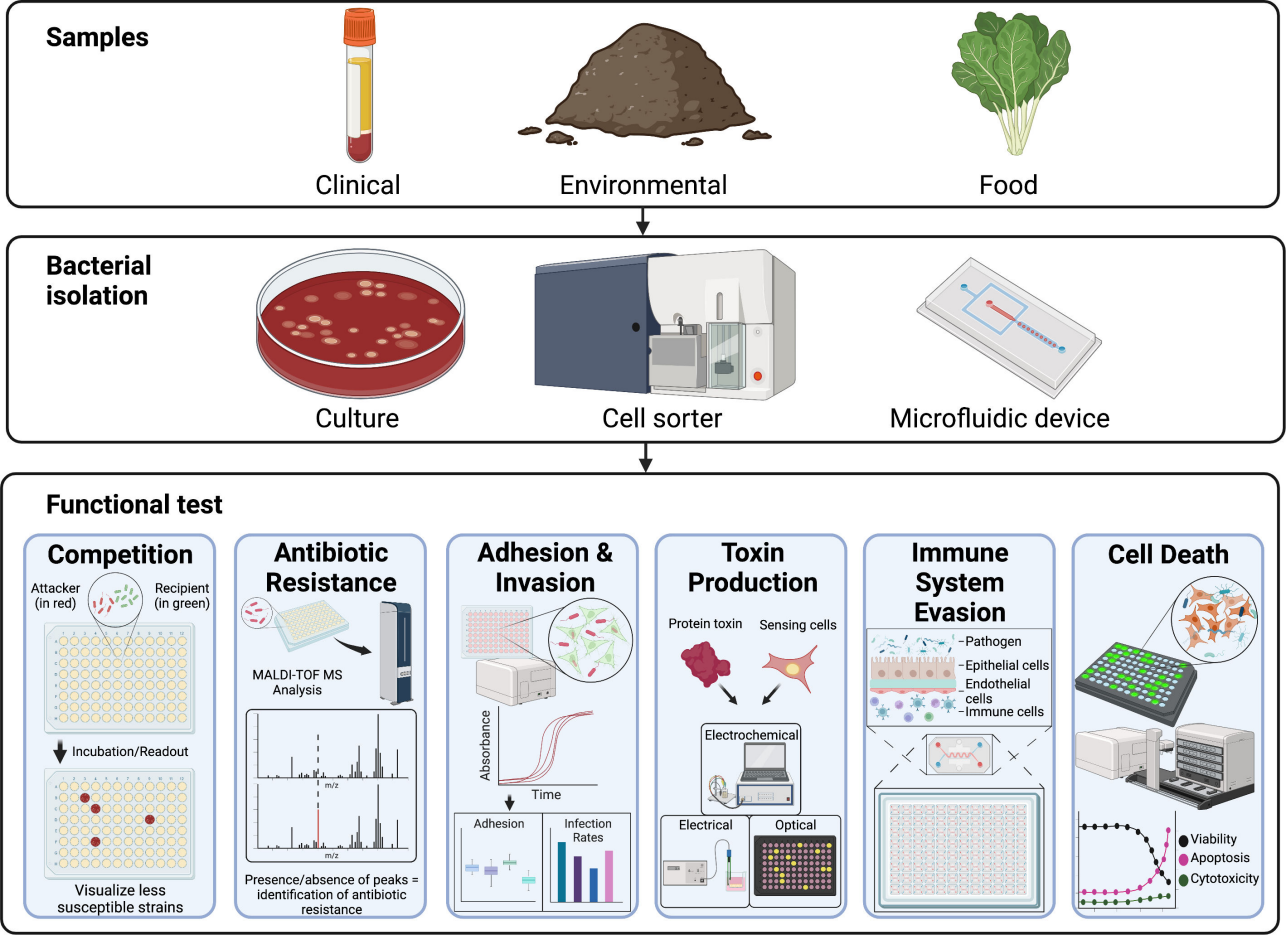
Six key functional traits of bacterial pathogens that are used during infection. Clinical, environmental, and food are common sample types for pathogen testing. From these sample types, pathogens can be isolated in pure culture on agar plates or by using devices such as cell sorters and microfluidic platforms. Methods for high-throughput trait testing utilize 96-well plates, liquid handlers, rapid detection systems (e.g., optical imaging and electrochemical), reporter cell lines, microfluidics, and miniature tissues. Pathogens use some or all traits to cause disease including, competition, antibiotic resistance, adhesion and invasion, toxin production, evasion of the immune system, and induction of cell death.

Over the past three decades, our ability to sequence DNA and RNA has increased exponentially (10). However, our ability to understand what the sequences infer is limited due to incomplete and constrained genome annotation. Furthermore, the physical, chemical, and biological dynamics that occur when a pathogenic bacterium, a conducive environment, and a susceptible host (e.g., plant, animal, and human) interact has not kept pace. Genes from newly sequenced genomes are typically annotated for function based on sequence similarity to characterized proteins, but the sheer number of possible proteins limits the accuracy of prediction. Databases now contain many proteins classified as hypothetical with unknown functions (11). A recent report from the U.S. Department of Energy’s Office of Biological and Environmental Research identified a need to “interrogate and characterize microbes and microbial communities at a scale and pace that matches genome sequence production” to enable predictive understanding of the behavior of newly discovered and emerging microbes (12). It would be a formidable challenge to characterize all functional traits of a pathogen. Therefore, in addition to genome annotation, validation of critical functional traits by high-throughput screens would improve our ability to link genomic information to pathogenic traits. Here, we describe some non-exhaustive examples of functional testing technologies that are providing insights into bacterial pathogens. Thus, this opinion article focuses on understanding recent advances in characterizing functional traits of pathogens and critical gaps in six key areas that are hallmarks of the infection process, including (i) competition, (ii) antibiotic resistance, (iii) adhesion and invasion, (iv) toxin production, (v) evasion of the immune system, and (vi) induction of cell death (Fig. 1).

## COMPETITION

During the introduction to a host, pathogens must use functional traits to outcompete existing microbes for nutrients and niches to establish infection and colonize host tissue. Competitive exclusion is a dominant principle by which existing microbes inhibit pathogen colonization (13). Depending on the ecosystem, several factors, such as the fitness of the current members, the niche, and arrival time, can influence competition between microbes (14). We compared the advantages and limitations of conventional laboratory, genetic, and high-throughput approaches for assessing competition and other functional traits as described in Table 1. Genetic methods such as genome-scale models enable the prediction of bacterial fitness and were used to analyze the phenotypic potential of *Escherichia coli* genotypes (15). Traditionally in the laboratory, competition has been measured using a few strains, often a single wild-type versus a single mutant strain, and reported as the competitive index (16). The traditional competitive index is a low-throughput assay calculated as the change in the ratio of the strains after mixture and growth together (17). Previous studies have used an *in vitro* approach to assess the fitness of *E. coli* strains over a few generations and up to 60,000 generations (18, 19). To increase the throughput, mutagenesis methods using transposon libraries with sequencing have been applied to multiple strains in competitive index assays and identified several genes required for virulence, functional redundancy, and functional independency of virulence factors (20). More recently, transposon-insertion sequencing has moved from a simple growth-based selection assay to an assessment of functional traits important for outcompeting other microbes on a scale of ~10^7^ mutant strains (21 – 23).

**TABLE 1:** Comparison of technologies for testing functional traits in bacterial pathogens

Trait	Assay type	Representative assay	Time to result (h)	Throughput	Resolves novel microbe or trait	Advantages	Limitations	Ref.
Competition	Conventional	*In vitro* fitness screen	24	12 Tests	Yes	Definitively identifies novel microbial traits	Does not identify the microbe; requires labor-intensive genetic modifications	(18)
	Genetic	Genome-scale models	168	One test	Yes	Definitively identifies novel microbial genetic traits	Does not predict microbial phenotype	(15)
	High throughput	Interbacterial competition screen	24	96 Tests	Yes	Definitively identifies novel microbial traits	Assay requires robotics which are expensive	(24)
Antibiotic resistance	Conventional	Disc diffusion	24	12 Tests	No	Rapid sample to answer	Does not identify the microbe	(25)
	Genetic	PCR Xpert (Cepheid)	6	96 Tests	No	Rapid sample to answer	Test panels do not identify engineered or emerging microbes	(26)
	High throughput	Direct-on-target microdroplet MALDI-TOF MS	≤6	96 Tests	No	Rapid sample to answer	Identification of the microbe is possible, but the microbial phenotype is not predicted	(27)
Adhesion and invasion	Conventional	*In vitro* adhesion and invasion assay	72	12 Tests	Yes	Definitively identifies novel microbial traits	Does not identify the microbe	(28)
	Genetic	Multiplex PCR for adhesins and invasins	6	96 Tests	No	Identifies phenotype of interest	Does not identify the microbe	(29)
	High throughput	Virtual colony counting infection assay	48	96 Tests	Yes	Definitively identifies novel microbial traits	Does not identify the microbe	(30)
Toxin production	Conventional	Toxin activity on cell monolayers	48	Six tests	Yes	Definitively identifies novel microbial traits	Does not identify the microbe	(31)
	Genetic	PCR of known toxin genes	6	96 Tests	No	Identifies phenotype of interest	Does not identify the microbe	(32)
	High throughput	Automated patch clamp platform	48	384 Tests	No	Identifies phenotype of interest	Does not identify the microbe	(33)
Immune system evasion	Conventional	Animal model	168	24 Tests	No	Identifies phenotype of interest	Labor and cost intensive; ethical concerns	(34)
	Genetic	Genome search for effectors tool	24	One test	No	Identifies phenotype of interest	Requires bioinformatics knowledge base; optimized for fungi; pipeline would need to be optimized for bacteria	(35)
	High throughput	Organ-on-a-chip	168	12 Tests	No	Identifies phenotype of interest	Labor intensive	(36)
Cell death	Conventional	Dye exclusion test	24	96 Tests	No	Rapid sample to answer	Does not identify the microbe	(37)
	Genetic	qPCR for apoptotic nucleic acids	6	96 Tests	No	Rapid sample to answer	Does not identify the microbe	(38)
	High throughput	Real-time fluorogenic DNA dyes	6	96 Tests	No	Rapid sample to answer	Does not identify the microbe	(39)

Competition and bacterial growth are often viewed to have a significant relationship. Atolia et al. found that noise minimization is critical for assessing growth by a high-throughput screen and that consistent growth when inoculating many cultures from bacterial colonies grown on agar plates is challenging (40). Automated robotic colony-picking systems may reduce the challenge of inoculating several cultures starting from bacterial colonies (41). To assess competition between microbes in a functional test, a high-throughput interbacterial competition assay enabling testing of 96 competition assays simultaneously was developed for *Agrobacterium tumefaciens* resulting in the observation that *A. tumefaciens* could kill other bacteria (24). This high-throughput interbacterial competition assay requires common laboratory materials such as 96-well plates but also uses an automated pipetting system (24) and could be expanded to other microbes. Other phenotypic high-throughput screening technologies, such as the Omnilog, have made it possible to investigate nearly 2,000 phenotypes related to nutrient competition (42). A limitation of these systems is that they measure the growth of the heterogeneous bacterial population. To overcome the issue of analyzing populations, novel methods based on microfluidic platforms have now made it possible to independently evaluate and track single cells on a scale of more than 10^5^ parallel cell lineages (43).

An additional consideration for high-throughput assessment is that the relationship between pathogenicity and growth may not be easily predicted. For example, when evaluating 61 human bacterial pathogens, the growth rate was negatively related to virulence (44). The growth rate may be considered a limitation for competition assays. Some bacterial pathogens are more amenable to high-throughput characterization because of growth characteristics, containment procedures, biosafety considerations, and ease of equipment sterilization between tests. To advance high-throughput competition assays, standardized systems should be developed that allow rapid assessment of multiple strains, standardized consortia of microorganisms relevant for different environments or host sites, and defined metrics for competition.

## ANTIBIOTIC RESISTANCE

Antibiotic resistance allows microbes to colonize environments with antibiotic stressors (45). In recent years, there has been an increase in infections caused by antibiotic-resistant bacteria (46). The interplay of bacterial virulence and antibiotic resistance is complex and depends on factors associated with the microbe and the environment (47). Traditionally, antibiotic susceptibility testing is performed using several cultivation rounds of a single isolate, and accepted breakpoint values are evaluated to determine whether the microorganism is susceptible or resistant (48). Recently, Yang et al. (49) developed a phenotype-based threat assessment pipeline characterizing bacterial pathogens for adherence, toxicity, immune activation, and antibiotic resistance. This previous study developed capabilities using machine learning with phenotypic data to assess pathogenic potential, and bacterial antibiotic resistance was assessed using traditional disk diffusion assays. Traditional methods such as disk diffusion assays can only test a few antibiotics (e.g., eight antibiotics) per agar plate and rely on isolating bacterial organisms in pure culture, which poses a bottleneck to antibiotic susceptibility testing (25). Thus, nucleic acid amplification and whole-genome sequencing (WGS) technologies are used in combination with functional testing. Commercial nucleic acid amplification kits exist for testing a wide range of antibiotics (26). However, caution should be used when interpreting genetic information as some studies have shown overall low sensitivity but high specificity for detecting antimicrobial resistance by nucleic acid amplification methods (50). Additionally, virulence plasmids containing antibiotic-resistant genes are being more widely reported, which can impact treatment and determine whether specific plasmids should be monitored to limit their spread (51, 52). Functional testing for antibiotic resistance can help link the antibiotic-resistant genes found on virulence plasmids.

Functional testing is critical to determine how bacterial organisms respond to new compounds, and which existing compounds are effective against novel strains. No single antimicrobial susceptibility testing technology is broadly accepted and globally accessible for rapid, high-throughput testing (53). In conjunction, there has been a lack of newly developed antibiotics despite the rise of robust screening methods for new drugs and drug combinations (54, 55). Although the use of artificial intelligence-driven discovery has drastically decreased the number of compounds needed to test (56), it remains challenging to test multiple compounds on several microorganisms simultaneously. Identification of effective compounds is critical because approximately 50% of antibiotic treatments begin with the wrong antibiotic without diagnosing the pathogen (48).

The traditional cultivation approach for testing the function of antibiotic resistance is time consuming and labor intensive. Thus, high-throughput methods are being developed to accelerate time-to-result and increase the diversity of cultivated bacterial organisms able to be tested. Recent advances in technology have relied on building devices using microfluidics and lab-on-a-disc systems with capabilities to test the growth of up to 100 bacterial strains within droplets (57 – 59). Another approach uses advances in matrix-assisted laser desorption/ionization time-of-flight mass spectrometry (MALDI-TOF MS) to rapidly screen functional sets of antibiotic resistance by direct-on-target microdroplet growth assays at a scale of 96 samples per assay (27, 60). The current challenge these assays face is increasing throughput and accessibility for routine testing. In addition, integrating different data types, such as MALDI-TOF MS and WGS, can be challenging. For integrating various data types, existing databases such as the Antimicrobial Testing Leadership and Surveillance, which provides open access to minimal inhibitory concentration, should be leveraged and integrated with genomic information (61).

## ADHESION AND INVASION

Adhesion is an important feature of bacterial pathogens that allows microbes to colonize hosts, induce cellular responses, and manipulate host signaling. To adhere to cells, bacteria use a range of factors, from pili, fimbria, flagella, and other adhesins (62 – 64). Conventional adherence assays are laborious and based on counting bacteria on agar plates or from stained cells (28, 65). Genetic methods, such as multi-plex PCR, test for the presence of multiple adhesin genes in a single reaction but do not provide evidence of functionality (29). Some high-throughput assays have been developed to quantify the extent of bacterial adhesion on host cells. For example, flow cytometry-based adherence assays that quantify interactions between bacteria and eukaryotic cells have been designed for fast and reproducible measurements (49, 66, 67). More recently, fluorescently tagged strains, bacteria carrying fluorescence proteins on plasmids, and live fluorescent stains have been used to quantify the level of adherence with a throughput of 96 samples per assay (49, 66, 67). Another method uses virtual colony counts, an absorbance-based measurement showing a good correlation with traditional plate-based colony counts for *Salmonella* with multiple eukaryotic cell lines (30). In addition, colony counting by high-throughput screens has enabled the determination of viable bacteria cell numbers in 96-well plates (68). Extensive washing to remove unbound bacteria and fixative treatment remains issues for high-throughput and traditional assays (66). To resolve finer-scale binding, host ligands can also be screened in high-throughput assays (69).

Certain pathogens also invade host cells and have an intracellular lifestyle. Along with conventional adherence assays, the laborious nature of invasion assays can be prohibitive to test multiple conditions and time points. Classically, the bacterial invasion has been quantified by a gentamicin protection assay in which internalized bacteria avoid being killed by gentamicin and can be enumerated (70). For rapid assessment, a screening system for invasion with a fluorescently tagged *Salmonella* strain was assessed using HeLa cells, enabling 24 samples to be tested per assay with applications for chemical libraries and potential drug testing (71). Similarly to adherence assays, the virtual colony counting high-throughput method has been applied to study *Salmonella* invasion, which can test 96 samples per assay (30). Tools that can characterize multiple functional traits, such as the virtual colony counting method, have greater utility.

## TOXIN PRODUCTION

Several bacterial pathogens use protein toxins to disrupt signaling, degrade biochemicals, and damage host tissue to establish infection (72). A plethora of toxins have been described in a variety of bacterial species. Toxins are not simply destructive tools but may contribute to survival and escape from environmental unicellular eukaryotes (73). Several methods exist for the specific detection of clinically important toxins, such as enzyme-linked immunoassay (ELISA), lateral flow tests, western blots, cell culture, and mass spectrometry (74). However, methods to simultaneously characterize known toxins, novel toxins, toxin potency, and integrate data from other functional traits and genomic information remain underexplored.

Traditionally, bacterial toxins were discovered and functionally characterized based on observing bacterial culture filtrates causing eukaryotic cell disruption or death (31). Although bacterial toxins are critical virulence factors, new toxins are continually being discovered. For example, the pore-forming toxin exolysin (ExlA) was initially described in 2014 from a virulent strain of *Pseudomonas aeruginosa* causing hemorrhagic pneumonia (75). For known toxins, selective agars containing chromogenic substrates that are cleaved by toxins have been developed to differentiate strains that produce active toxins from non-producing strains (32). The throughput of using chromogenic media is often limited to one strain per agar plate and can be time consuming based on the incubation time of the bacterial strains. In conjunction, genetic methods such as PCR are often used for follow-up testing to characterize and subtype toxins which can increase the time-to-result. Also, PCR often does not provide an independent result for function but rather only tests for the presence of the gene (32). To our knowledge, high-throughput screens have primarily been developed for chemical toxins and not as robustly for bacterial toxins (76, 77). However, automated patch clamp platforms exist that measure ionic current and the state of voltage-gated ion channels (e.g., open or closed) that bacterial toxins often act upon and can be used for functional measurements. Automated patch clamp platforms such as the SyncroPatch is a high-throughput platform that can assess 384 and 768 samples and was applied to detecting tetrodotoxin produced by pathogenic bacteria such as *Pseudomonas* and *Vibrio* species (33). Although the automated patch clamp throughput is considerably higher than selective media, the construction and stable expression of channels in reporter cell lines that the bacterial toxins act on are limiting factors.

## EVASION OF THE IMMUNE SYSTEM

Many pathogens have developed defenses to evade the host innate immune system. Some pathogenic bacteria directly inject proteins termed effectors into target host cells via specialized secretion systems across the bacterial and host membranes to manipulate host cell functions. One of the best characterized secretion systems is the type III secretion system (T3SS) (78). T3SSs inject effector proteins that are diverse and specific to pathogens to induce pathogenic mechanisms, specifically immune system subversion. Pathogens use T3SSs effectors to evade host immunity in several ways, such as activating host-signaling cascades and pattern-recognition receptors, and suppressing evasion of innate and adaptive defenses (79).

There is a need for efficient methods to identify effectors in pathogenic bacteria. The Genome Search for Effectors Tool (GenSET) can predict effector sequences in bacteria (35). This tool can provide information for researchers to conduct downstream wet bench experimental validation; however, GenSET has low prediction rates. This could be caused by the various families of T3SSs found in different species, as heterologous effectors may yield other attributes when applied to specific microbes. Low prediction rates can provide inaccurate results, hence, the need for downstream validation (35). Improved computational approaches and machine-learning algorithms are needed to identify novel effectors from unannotated nucleotide and protein sequences accurately.

In the laboratory, animal models are often used to test the ability of bacterial factors to evade the innate immune system (34). In contrast, *in vitro* platforms are more cost-effective and rapid. There are several reporter cell lines for detecting immune system activation such as the HEK-Blue TLR2, TLR3, TLR4, TLR5, TLR7, TLR8, and TLR9 cells (InvivoGen) or the nuclear factor κB reporter line used previously (49). For the detection of immune system evasion, it is vital to characterize bacterial pathogens in relevant *in vitro* systems such as co-culture of epithelial and immune cells. Recent studies investigated “gut-on-chips” to simulate structure and function as an attempt to replicate *in vivo* microenvironments (80). These tissue chips are a promising approach for studying microbe-host interactions in a more high-throughput screen than animal testing. They can incorporate mononuclear phagocytes to respond to commensal or pathogenic bacteria in a 3D configuration. A challenge with co-culturing cell types with different tissues is that the optimum conditions for each cell type may differ, leading to inaccurate real-time interactions. Other challenges include immune cell adherence, material compatibility, selection of extracellular matrix (ECM), and immune cell migration through ECM (81). However, organ-on-chip devices provide a promising avenue of research for understanding epithelial and immune cell responses to potential pathogens.

## INDUCTION OF CELL DEATH

During infection, host cells will respond to a pathogen in various ways, including cell death, to remove the infected cell from the host. Cell death has regulated pathways to initiate death and various morphological and molecular changes in the cell characterize each pathway. Some common mechanisms for cell death include apoptosis, necrosis, and pyroptosis, with the most characterized being apoptosis (82). Apoptosis is a non-inflammatory programmed cell death type characterized by changes in cell morphology, such as membrane blebbing, cell shrinkage, and DNA fragmentation. Banfalvi reviewed several assays that detect apoptosis related to structural and functional changes (83). In addition, non-programmed cell death can occur from infection (84).

Cell death assays have been developed for high-throughput screens. Cummings et al. reviewed these methodologies in detail (85). However, most assays have been designed from a drug discovery perspective rather than to assess cell death from pathogens. Drug discovery assays can determine cell death and what pathways are activated (85), but they should be more widely considered for characterizing bacterial pathogens. In addition to antibiotic resistance, immune activation, and adherence, the platform developed by Yang et al. (49) tested bacterial-induced cell death by using cell staining and flow cytometry.

Pathogenic bacteria can activate several cellular pathways. We previously engineered a fluorescent reporter lung cell line that signals when the protein kinase ERK (extracellular signal-regulated kinase) and transcription factor Fra1 (FOS-related antigen 1) pathway are activated or inhibited (86). The change in fluorescent signal occurs before the stress response/cytopathic effects of the host cell. This high-throughput image-based assay allows for functional screening of cell health with the incubation of a live bacterial strain. A limitation of this assay is that not all pathogenic bacteria can inhibit the ERK-Fra1 signaling pathway. There is an opportunity to increase the number of fluorescent reporter pathways for cell death and cell types to include a broader range of pathogens using this approach (86).

## LIMITATIONS AND CONCLUDING REMARKS

Linking and integration of genotype to phenotype will enhance pathogen characterization. The amount of genetic sequence data so far surpasses functional trait data for bacterial pathogens. Thus, high-throughput functional assays are needed to keep pace with genetic sequence data. Recently, PathEngine used an integrated strategy of a phenotype-based pipeline to assess pathogenic potential, which included adherence, toxicity, antibiotic resistance, and innate immune activation (49). A significant hurdle with harnessing functional assay testing data is integrating information generated from these assays with existing information such as sequence, transcriptomic, proteomic, and metabolomic data. To harness this data, robust and agile databases are needed. As proposed previously, multiomics integration to identify pathogen-agnostic signatures of disease could detect potential pathogens without prior knowledge of the microbe (87).

Another limitation is that the integration of pathogen functional data is challenging because it can be unclear how individual proteins work as part of a global infection model. Therefore, integrating multiple bacterial functional traits to understand virulence has not been robustly developed. Developing strategies to use multiple pieces of information, such as WGS, proteomics, and the functional tests discussed in this work, will give rise to a more detailed understanding of classifying known and novel organisms for their pathogenic potential.
